# COVID-19 outbreak in an acute psychiatric unit—Unique challenges and creative solutions

**DOI:** 10.1017/ash.2023.361

**Published:** 2023-09-29

**Authors:** Supriya Narasimhan, Sherilyn Oribello, Laura Tang, Tracey Stoll, Vidya Mony

## Abstract

**Background:** We describe the management of a major COVID-19 outbreak in January 2022 during the SARS-CoV-2 omicron-variant winter surge involving the only inpatient psychiatric facility of Santa Clara County, California, which serves a population of 1.9 million. **Methods:** On January 14, 2022, infection prevention staff were notified of a symptomatic COVID-19 case in our locked inpatient psychiatric unit who had been admitted since October 2021. The index patient had no visitors or transfers outside the unit. The patients in this unit were noncompliant with masking and mingled with each other during meals. Initial testing identified 23 positive cases among 47 patients and 12 staff cases. Mitigating actions included closing the unit to new admissions, creating alternate care areas in the emergency psychiatric unit, and separating patients into “exposed but negative” and “infected” cohorts and housing them in “red,” and “yellow” zones, respectively. A “green” zone was created by clearance of positive cases. For the cohort exposed to COVID-19, masking was enforced by supervision, dining was scheduled in batches, and daily symptom screening and antigen testing were performed in addition to standard postexposure RT-PCR testing on day 4 and day 7. Mandatory N95 respirators and eye protection were implemented for staff on unit entry. Exposed staff followed employee health protocols for postexposure testing. Enhanced environmental control measures included terminal cleaning and UV-C disinfection of common areas and patient rooms and a thorough investigation of airflow. Discharged patients were contacted if they were residing in congregate facilities. **Results:** Of 47 patients, 39 (83%) tested positive for COVID-19. However, 8 patients remained negative; all 8 had received at least their primary vaccine series (Table 1). In total, 16 HCWs were SARS-CoV-2 positive in this outbreak. The outbreak officially ended 25 days after the first case. All SARS-CoV-2–positive patients had mild illness, not requiring treatment or hospitalization. We identified vaccine immune escape, staff presenteeism, patient noncompliance with masking, and comingling as major causes of transmission. We determined through contact tracing and temporality that the outbreak likely started from a positive staff member or visitor because most patients had been long-term residents. **Conclusions:** This outbreak was challenging due to the specialized behavioral needs of the involved patients. It was imperative to reopen this unit quickly and safely to provide psychiatric care to our county’s most vulnerable patients. Ongoing PPE education, repeated reinforcement, engagement in staff wellness to combat pandemic fatigue, and aggressive vaccination are all crucial to minimizing the impact of future outbreaks.

**Figure:**
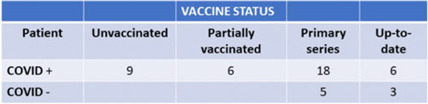

**Disclosures:** None

